# Somatic amplification and addiction profile as risk factors for medication overuse headache with chronic migraine

**DOI:** 10.1007/s10072-024-07639-w

**Published:** 2024-06-13

**Authors:** Ender Cesur, Burcu Göksan Yavuz, Erkan Acar, Zeynep Özdemir, Tuba Erdoğan Soyukibar, Elif Ilgaz Aydınlar

**Affiliations:** 1 Department of Psychiatry, Acibadem Mehmet Ali Aydinlar University Medical Faculty, Istanbul, Türkiye; 2 Department of Neurology, Acibadem Mehmet Ali Aydinlar University Medical Faculty, Istanbul, Türkiye; 3 Department of Neurology, Bakirkoy Prof Mazhar Osman Training and Research Hospital, Istanbul, Türkiye

**Keywords:** Addiction, chronic migraine, medication-overuse headache, somatic amplification

## Abstract

**Introduction:**

Overuse of analgesics can lead to medication-overuse headache (MOH) in chronic migraine (CM) patients, and is often linked to addiction. This study explores the addiction-related characteristics and somatic amplification in patients with, CM with medication overuse headache (CM+MOH), CM, and healthy controls.

**Methods:**

73 CM patients and 70 CM+MOH, along with 63 healthy controls, participated in the study. Assessments included a Sociodemographic Form, Migraine Disability Assessment Scale (MIDAS), Addiction Profile Index (API), Addiction Profile Index-Clinical Version (API-C), and the Somatosensory Amplification Scale (SSAS).

**Results:**

Substance use characteristics, craving, motivation for use, and addiction severity scores were higher in the CM+MOH group than in both the CM and the control group. Specifically, the SSAS scores within the CM+MOH group surpassed those of both the CM and control groups. In the CM+MOH group, SSAS scores were a strong predictor of the amount of analgesic usage. Besides, craving and motivation for substance use scores significantly predicted the number of days analgesic taken per month in the CM+MOH group

**Conclusion:**

CM patients with MOH exhibit a pronounced association with addiction, and a heightened manifestation of somatic symptoms. Addressing addiction characteristics and psychosomatic amplification is important to ensure comprehensive management.

## Introduction

Chronic migraine (CM) is defined as having a headache on at least 15 days per month for more than three months, with the features of migraine headache present on at least eight days per month [, 1,2**]**. Patients with CM have a high frequency of comorbid psychiatric disorders [3, 4]. Psychiatric comorbidities are related to disease prognosis and clinical outcomes. Some psychiatric comorbidities, including depression, are associated with worsening disease, as seen in the progression from episodic to CM and disease outcomes, including suicide attempts [3, 5]. Previous studies have suggested an association between migraine and an increased risk of substance abuse and addiction [6, 7]. Individuals experiencing migraine attacks may resort to substance use as a coping mechanism for pain management [7, 8]. Additionally, migraine-related psychosocial effects may contribute to increased susceptibility to addiction, especially in individuals trying to cope with emotional stressors [8]. Migraine patients may also tend to overuse painkillers excessively due to pain. Medication overuse headache (MOH), a secondary headache disorder, often complicates the treatment of primary headache disorders and other secondary headache disorders [9]. MOH is the term applied to headache occurring on 15 or more days per month developing as a consequence of regular overuse of acute headache medication for more than three months. This includes ergotamines, triptans, opioids, or combination analgesics, or any combination of ergotamines, triptans, simple analgesics, nonsteroidal anti-inflammatory drugs and/or opioids without overuse of any single drug or drug class alone for ≥10 days per month for >3 months or simple analgesics for ≥15 days per month for >3 months [1]. The general population's prevalence of MOH is around 1 to 2 percent in most studies, with a higher incidence in females than males [10]. Migraine is the most common primary headache disorder associated with MOH [11]. The exact processes contributing to MOH remain unclear. Nevertheless, various factors appear to be involved, including genetic predisposition, central sensitization, and biobehavioral factors [12].

A longitudinal study in Norway involving 32,067 adults revealed that those using analgesics daily or weekly at baseline had a significantly higher risk of developing chronic headaches 11 years later, with the highest risk observed in individuals with chronic migraine [13]. In a case-control study comparing patients with MOH derived from migraine to those with episodic migraine, the former group showed a significantly greater risk of substance-related disorders. In addition to medication overuse, anxiety and depression are identified as potential risk factors for the progression of migraine into MOH [14].

While research has established a connection between migraine and psychiatric disorders [, 14,15**]**, there is limited understanding of the link between somatic amplification and migraine-related disability [16]. Somatosensory amplification refers to the inclination to interpret normal somatic and visceral sensations as disproportionately intense, unpleasant, and harmful. This tendency is commonly associated with hypochondriasis and is frequently observed in conjunction with conditions such as fibromyalgia, major depressive disorder, and certain anxiety disorders [17]. It posits that individuals prone to somatization perceive ordinary bodily sensations as unusually intense and distressing. Psychological factors and heightened sensitivity to bodily sensations may contribute to the overall burden of migraine. Yavuz et al. (2013) point out that timely assessing somatic amplification and evaluating mental status would help improve the quality of life of migraineurs [16]. However, no studies have been found on somatic amplification in migraine patients diagnosed with MOH [17].

The aim of this study is to investigate the addiction-related clinical characteristics, and somatic amplifications of patients with CM+MOH, CM, and healthy control groups, and to contribute to clinicians for treatment. We hypothesize that somatic amplification may increase the medication overuse in migraineurs. The second hypothesis of this study is that substance use and addiction are common in patients diagnosed with CM+MOH and that these are predictors of medication overuse.

## Methods

### Participants

From January 2022 to October 2023, 147 out of 216 patients diagnosed with CM at the outpatient unit agreed to participate in the study. Among these, 143 patients (consisting of 73 with CM and 70 with CM+MOH) who completed the forms were included. CM was diagnosed by two neurologists based on the International Classification of Headache Disorders diagnostic criteria that require headache occurring on ≥15 days per month for >3 months with at least five attacks fulfilling criteria of migraine without aura on ≥8 days per month [2]. Although other primary headaches can cause MOH , we only included patients with MOH with CM in the study because we aimed to reveal some risk factors for the development of MOH in CM patients. These patients were cited as "CM+MOH” in this study.

Exclusion criteria were episodic migraine, acute and chronic psychosis, mental retardation and illiteracy. Sixty-three healthy control subjects matched in age and sex were recruited. The control subjects did not meet the criteria for migraine, and other primary and secondary headaches.

After the patients gave written informed consent, the neurologists questioned their sociodemographic and clinical characteristics. Then, the subjects were asked to fill out a Migraine Disability Assessment Scale (MIDAS), Addiction Profile Index (API), Addiction Profile Index-Clinical Version (API-C), and the Somatosensory Amplification Scale (SSAS). Participants in the control group were also asked to fill out forms other than MIDAS.

The study protocol was conducted per the ethical principles stated in the “Declaration of Helsinki” and approved by the Ethical Committee of our university (17.12.2021;2021/24).

### Measures

#### Sociodemographic and Clinical Evaluation Form

The researchers prepared a questionnaire that included questions about sociodemographic and clinical characteristics. Migraine onset date, duration, attack severity, psychiatric and medical history, and medication use characteristics were recorded.

#### Migraine Disability Assessment Scale (MIDAS)

The evaluation of migraine-associated disability utilized the validated Turkish adaptation of the MIDAS questionnaire. This assessment comprises five inquiries aimed at quantifying the number of workdays forfeited due to migraines within a three-month span. MIDAS captures data on the disability stemming from migraines in relation to work/study, household work, and leisure activities when the headache was experienced during headache episodes [18]. The questions inquire about either missed activity days or days where productivity was diminished by at least 50%. The cumulative total of these days is then categorized into four severity grades. MIDAS A and MIDAS B assess headache frequency and pain intensity (0= no pain; 10= very severe pain) over three months. The Turkish version of the MIDAS questionnaire was developed by Ertas et al. [19].

#### The Somatosensory Amplification Scale (SSAS)

The Somatosensory Amplification Scale (SSAS) is designed to assess sensitivity to mild bodily sensations that are unpleasant and distressing but non-pathological. Developed by Barsky et al., this self-report scale comprises 10 items, each rated on a five-point scale ranging from 1 (not at all) to 5 (extremely) [17]. The statements describe physical discomfort that does not suggest the presence of a disease. The sum of these scores yields a total amplification score. Gülec et al. have established the reliability and validity of the Turkish version of this scale [20].

#### Addiction Profile Index (API)

The Addiction Profile Index (API), developed by Ögel et al. (2012), is a self-report questionnaire that consists of 37 items to evaluate substance use characteristics and severity [21]. Each item is rated from 0 to 4 on a five-point Likert scale. The questionnaire consisted of five subscales measuring the characteristics of substance use, diagnostic criteria, effect on everyday life, craving, and motivation for substance use.

#### Addiction Profile Index-Clinical Version (API-C)

The addiction level was evaluated by the Addiction Profile Index-Clinical Version (API-C) developed by Ögel et al. (2015) [22]. Participants underwent the API-C scale to gather information on their current substance use patterns and the mental and personal aspects of substance use during probation. The API-C encompasses the evaluation of six dimensions that persist and coexist with addiction, extending beyond those directly associated with addiction itself. These dimensions include depression, anxiety, anger control failure, lack of safe behavior, novelty-seeking behavior, and impulsivity. The scale comprises 58 items; scores below 12 points indicate a low level of addiction, scores between 12 and 14 points suggest a moderate level of addiction, and scores above 14 points indicate a high level of addiction.

### Statistics

SPSS (version 26) program was used for statistical data analysis. One-way analysis of variance (ANOVA) was used to compare continuous variables between three groups, and an independent sample t-test was used to compare two patient groups. The chi-square test was used to compare groups in terms of categorical variables. Multiple regression analysis was performed to determine the variables that predicted the monthly amount and days of analgesic use in the patient groups. In the regression analysis, number of days analgesic taken per month and monthly analgesic use amount as the dependent variables, API, API-C and the SSAS scores as independent variables were added to the model at once, and variables that did not contribute to the model were excluded using the backward elimination method. In variance analyses, effect sizes were presented as partial eta squared (*η*_*p*_^*2*^), and in t-test analyses, effect sizes were presented as Cohen's *d*. Statistical significance was accepted as *p*< 0.05 in all analyses.

## Results

### Demographic Characteristics

Demographic characteristics of the control, CM+MOH, and CM groups were compared.

#### Formun Üstü

The CM+MOH group showed a higher occurrence of psychiatric history (48.6%) compared to both the control group (15.9%) and CM group (16.4%) (χ2 (2) = 24.506, *p*< 0.001). Additionally, the working rates in the control group (85.7%) were found to be higher than those of both patient groups (χ2 (2) = 9.887, *p* = 0.007). The rate of medical history was higher (χ2 (2)= 9.907, *p*= 0.007) in the CM+MO group than in the control group and the CM group (Table 1).

**Table 1:** Comparison of demographic characteristics of the control group and patient groups

	Control (*n*= 63)	CM (*n*= 73)	CM+MOH (*n*=70)	Statistics	*p*
Age, Mean ± SD	35.06 ± 5.57	35.41 ± 7.40	35.61 ± 9.65	*F*(2, 205)= 0.084	0.919
Sex				*χ*^*2*^ (2)= 0.023	0.988
Female	54 (%85.7)	62 (%84.9)	60 (%85.7)		
Male	9 (%14.3)	11 (%15.1)	10 (%14.3)		
Education				*χ*^*2*^ (2)= 2.290	1.000
Primary school	0	1 (%1.4)	0		
Middle school	1 (%1.6)	1 (%1.4)	1 (%1.4)		
High school	6 (%9.5)	7 (%9.6)	6 (%8.6)		
University and above	56 (%88.9)	64 (%87.7)	63 (%90.0)		
Marital Status				*χ*^*2*^ (2)= 6.658	0.838
Married	22 (%34.9)	32 (%43.8)	29 (%41.4)		
Single	36 (%57.1)	39 (%53.4)	37 (%52.9)		
Estranged	1 (%1.6)	1 (%1.4)	0		
Divorced	3 (%4.8)	1 (%1.4)	2 (%2.9)		
Widow	1 (%1.6)	0	1 (%1.4)		
Other	0	0	1 (%1.4)		
Having Children				*χ*^*2*^ (2)= 0.140	0.934
Yes	23 (%36.5)	26 (%35.6)	27 (%38.6)		
No	40 (%63.5)	47 (%64.6)	43 (%61.4)		
Psychiatric History				*χ*^*2*^ (2)= 24.506	< 0.001
Yes	10 (%15.9)	12 (%16.4)	34 (%48.6)		
No	53 (%84.1)	61 (%83.6)	36 (%51.4)		
Working Status				*χ*^*2*^ (2)= 9.887	0.007
Unemployed	9 (%14.3)	22 (%30.1)	27 (%38.6)		
Employed	54 (%85.7)	51 (%69.9)	43 (%61.4)		
Medical History				*χ*^*2*^ (2)= 9.907	0.007
Yes	8 (%12.7)	16 (%21.9)	25 (%35.7)		
No	55 (%87.3)	57 (%78.1)	45 (%64.3)		

*CM* Chronic migraine, *MOH* Medication overuse headache, *SD* Standard deviation

### Clinical Characteristics of CM+MO and CM Groups

Comparing the CM+MOH and CM groups in terms of clinical characteristics revealed significant differences. The duration of migraine chronification (*t*(141)= 2.907, *p*< 0.001, *d*= 0.49), duration of migraine disease (*t*(141)= 4.138, *p*< 0.001, *d*=0.70), migraine attack severity (*t*(141)= 6.342, *p*< 0.001, *d*=1.07), number of days analgesic taken per month (*t*(141)= 9.444, *p*< 0.001, *d*= 1.57), amount of total analgesics taken in a month (tablets) (*t*(141)= 7.366, *p*< 0.001, *d*= 1.22), amount of migraine-specific analgesics taken in a month (tablets) (*t*(141)= 6.470, *p*< 0.001, *d*= 1.08) and amount of non-spesific analgesics taken in a month (tablets) (*t*(141)= 5.949, *p*< 0.001, *d*= 0.99) were significantly higher in the CM+MOH group than the CM group. The number of current prophylactic agents was higher in the CM+MOH group than in CM group (*χ*^*2*^ (2)= 18.803, *p*< 0.001) and the response rates to analgesics were lower compared to the CM group (*χ*^*2*^ (2)= 41.165, *p*< 0.001) (Table 2).

**Table 2:** Clinical features of patient groups

	CM (*n*= 73)	CM+MOH (*n*=70)	Statistics	*p*	*d*
Migraine disease duration(year), Mean ± SD	9.08 ± 5.86	12.51 ± 8.12	*t*(141)= 2.907	0.004	0.49
Migraine attack duration (hour), Mean ± SD	12.78 ± 7.50	20.61 ± 14.25	*t*(141)= 4.138	< 0.001	0.70
Headache severity, Mean ± SD	6.12 ± 1.58	7.77 ± 1.52	*t*(141)= 6.342	< 0.001	1.07
Current prophylactic agents			*χ*^*2*^ (2)= 18.803	< 0.001	-
Yes	15 (%20.5)	39 (%55.7)			
No	58 (%79.5)	31 (%44.3)			
Number of days analgesics use (month), Mean ± SD	8.41 ± 4.50	17.05 ± 6.33	*t*(141)= 9.444	< 0.001	1.57
Number of analgesics a month (tablets), Mean ± SD	11.15 ± 10.18	31.67 ± 21.42	*t*(141)= 7.366	< 0.001	1.22
Number of migraine-specific analgesics in a month (tablets), Mean± SD	3.00 ± 5.69	11.13 ± 9.02	*t*(141)= 6.470	< 0.001	1.08
Number of nonspesific analgesics in a month (tablets), Mean± SD	8.15 ± 5.92	21.19 ± 17.72	*t*(141)= 5.949	< 0.001	0.99
Response to analgesics			*χ*^*2*^ (2)= 41.165	< 0.001	-
No response	0	3 (%4.3)			
Little	3 (%4.1)	21 (%30.0)			
Moderate	15 (%20.5)	29 (%41.4)			
Good	35 (%47.9)	12 (%17.1)			
Very good	20 (%27.4)	5 (%7.1)			

*CM* Chronic migraine, *MOH* Medication overuse headache, *SD* Standard deviation

### Comparison of Control and Patient Groups in Terms of Research Variables

The control and patient groups were compared in terms of API, substance use characteristics (F(2, 205)= 33.081, *p*< 0.001, *η*_*p*_^*2*^= 0.25), diagnostic criteria F(2, 205)= 41.050, *p*< 0.001, *η*_*p*_^*2*^= 0.29), effects on everyday life (F(2, 205)= 33.193, *p*< 0.001, *η*_*p*_^*2*^= 0.27), craving (F(2, 205)= 30.534, *p*< 0.001, *η*_*p*_^*2*^= 0.23), motivation for substance use (F(2, 205)= 32.600, *p*< 0.001, *η*_*p*_^*2*^= 0.24) and total addiction severity (F(2, 205)= 46.454, *p*< 0.001, *η*_*p*_^*2*^= 0.31) and the scores were found to differ between groups. The post-hoc analyses, performed to search for the source of these differences, revealed that all CM+MOH group scores were higher than the CM and the control group (Bonferroni correction applied in all comparisons, *p* < 0.001). There was no significant difference between the API scores of the control and the CM groups (Table 3).

**Table 3:** Addiction Profile Index (API), API Clinical Form (API-C), Somatosensory Amplification Scale and Suicide Behavior Questionnaire scores comparison between control and patient groups

	Control (*n*= 63)	CM (*n*= 73)	CM+MOH (*n*=70)	Statistics	*p*	*η*_*p*_^*2*^
API – Substance use characteristics	0.04 ± 0.07	0.06 ± 0.14	0.69 ± 0.91	*F*(2, 205)= 33.081	< 0.001	0.25
API – Diagnostic criteria	0.10 ± 0.39	0.18 ± 0.64	2.98 ± 3.57	*F*(2, 205)= 41.050	< 0.001	0.29
API– Effect on everyday life	0.13 ± 0.73	0.33 ± 1.11	4.59 ± 5.66	*F*(2, 205)= 38.193	< 0.001	0.27
API – Craving	0.00 ± 0.00	0.12 ± 0.47	1.86 ± 2.63	*F*(2, 205)= 30.534	< 0.001	0.23
API – Motivation for substance use	0.03 ± 0.25	0.07 ± 0.25	1.97 ± 2.75	*F*(2, 205)= 32.600	< 0.001	0.24
Total addiction severity	0.09 ± 0.29	0.21 ± 0.59	3.42 ± 3.91	*F*(2, 205)= 46.454	< 0.001	0.31
API-C– Anger control failure	2.11 ± 1.60	1.55 ± 1.46	3.04 ± 1.88	*F*(2, 205)= 14.827	< 0.001	0.13
API-C – Lack of safe behavior	2.62 ± 2.18	2.85 ± 2.53	5.09 ± 2.88	*F*(2, 205)= 19.504	< 0.001	0.16
API-C – Novelty-seeking behavior	1.48 ± 1.46	1.41 ± 1.60	2.61 ± 1.81	*F*(2, 205)= 11.938	< 0.001	0.11
API-C – Impulsivity	1.19 ± 1.24	1.58 ± 1.64	3.06 ± 1.78	*F*(2, 205)= 26.483	< 0.001	0.21
API-C – Depression	1.30 ± 1.41	1.68 ± 1.71	3.77 ± 2.25	*F*(2, 205)= 36.029	< 0.001	0.26
API-C - Anxiety	0.81 ± 1.08	1.16 ± 1.40	2.57 ± 1.82	*F*(2, 205)= 27.130	< 0.001	0.21
Somatosensory Amplification Scale	15.08 ± 3.92	16.14 ± 4.42	31.23 ± 7.91	*F*(2, 205)= 171.221	< 0.001	0.63

*CM* Chronic migraine, *MOH* Medication overuse headache

When API-C scores were calculated, anger control failure (F(2, 205)= 14.827, *p*< 0.001, *η*_*p*_^*2*^=0.13), lack of safe behavior (F(2, 205)= 19.504, *p*< 0.001, *η*_*p*_^*2*^= 0.16), novelty-seeking behavior (F(2, 205)= 11.398, *p*< 0.001, *η*_*p*_^*2*^= 0.11), impulsivity (F(2, 205)= 26.483, *p*< 0.001, *η*_*p*_^*2*^= 0.21), depression (F(2, 205)= 36.029, *p*< 0.001, *η*_*p*_^*2*^= 0.26) and anxiety (F(2, 205)= 27.130, *p*< 0.001, *η*_*p*_^*2*^= 0.21) scores differed between the three groups. Post-hoc analyses, revealed that all scores of the CM+MOH group were higher than the CM and the control group (Bonferroni correction was applied in the comparison of the control group and the CM group in API-C anger control failure scores; *p* = 0.004 for all other groups). Bonferroni correction was applied in comparisons (*p*< 0.001). There was no significant difference between the API-C scores of the control and CM group (Table 3).

The comparison of SSAS scores between the three groups, showed that the scores of both scales were significantly different between the three groups (F(2, 205)= 171.221, *p*< 0.001, *η*_*p*_^*2*^= 0.63). The SSAS scores of the CM+MOH group were higher than the CM and control group in post-hoc analysis (Bonferroni correction applied *p*< 0.001). No significant difference was detected between the control and CM group in both scale scores (Table 3).

### Comparison of migraine disability assessment scale (MIDAS) scores

MIDAS total scores (*t*(141)= 8.242, *p*< 0.001, *d*= 1.39), MIDAS-A scores (*t*(141)= 8.651, *p*< 0.001, *d*= 1.46) and MIDAS-B scores (*t*(141)= 8.651, *p*< 0.001, *d*= 1.46) of the CM+MO group were found to be statistically significantly higher than the scores of the CM group (Table 4).

**Table 4:** Migraine Disability Assessment Scale (MIDAS) scores comparison between patient groups

	CM (*n*= 73)	MOH (*n*=70)	Statistics	*p*	*d*
MIDAS total	25.12 ± 19.33	63.67 ± 37.75	*t*(141)= 8.242	< 0.001	1.39
MIDAS-A	20.55 ± 10.65	43.27 ± 19.63	*t*(141)= 8.651	< 0.001	1.46
MIDAS-B	6.25 ± 1.65	8.06 ± 3.66	*t*(141)= 3.845	< 0.001	0.65

*CM* Chronic migraine, *MOH* Medication overuse headache

### Variables predicting the amount of analgesics used per month in patient groups

Craving among the API subscales (*β*= -0.646, *p*= 0.021) and the SSAS scores (*β*= 0.250, *p*= 0.030), significantly predicted the amount of analgesic use in the CM group. And the model consisting of these two variables was significant *(F*(2, 72)= 3.263, *p*= 0.027) Only the SSAS scores (*β*= 0.381, *p*= 0.001), among the variables added to the model, significantly predicted the amount of analgesic use in the CM+MOH group (F(1, 69)= 11.558, *p*= 0.001) (Table 5).

**Table 5:** Variables predicting the number of analgesics used per month in patient groups

Group	Variables	*β*	t	*p*	Statistics
CM	API – Craving	-0.646	-2.368	0.021	R= 0.35; R^2^= 0.12; Dzt. R^2^= 0.09; F(2, 72)= 3.263, *p*= 0.027
	Somatosensory Amplification Scale	0.250	2.212	0.030	
CM+MOH	Somatosensory Amplification Scale	0.381	3.400	0.001	R= 0.38; R^2^= 0.15; Dzt. R^2^= 0.13; F(1, 69)= 11.558, *p*= 0.001

*CM* Chronic migraine, *MO* Medication overuse headache

### Variables predicting the number of days analgesic taken per month in patient groups

Craving among the API subscales (*β*= -0,233, *p*= 0,039) and the API-C– Lack of safe behavior scores (*β*= 0,381, *p*= 0,006), significantly predicted the number of days analgesic taken per month in the CM group. Moreover the model consisting of these two variables was significant *(F*(2, 72)= 3,659, *p*= 0,009). Besides, craving (*β*= -0,393, *p*= 0,031) and motivation for substance use scores (*β*= 0,465, *p*= 0,009) significantly predicted the number of days analgesic taken per month in the CM+MOH group (F(2, 69)= 2,703, *p*= 0,038) (Table 6).

**Table 6:** Variables predicting the number of days analgesic taken per month in patient groups

Group	Variables	*β*	t	*p*	Statistics
CM	API– Craving	-0.233	-2.101	0.039	*R*= 0.42; *R*^*2*^= 0.18; *Adj. R*^*2*^= 0.13; *F*(2, 72)= 3.659, *p*= 0.009
	API-C – Lack of safe behavior	0.381	2.838	0.006	
CM+MOH	API– Craving	-0.392	-2.200	0.031	*R*= 0.38; *R*^*2*^= 0.14; *Adj. R*^*2*^= 0.09; *F*(2, 69)= 2.703, *p*= 0.038
	API– Motivation for substance use	0.465	2.700	0.009	

*CM* Chronic migraine, *MOH* Medication overuse headache, *Adj.* Adjusted

## Discussion

The present study investigated the addiction-related clinical characteristics, and somatic amplifications of patients with CM+MOH, CM, and healthy control groups, and the parameters that predict the amount of analgesic use in the patients.

Headache severity, number of days analgesics taken per month, amount of analgesics taken, and the amount of current prophylactic agents in the CM+MOH group were higher than in the CM group. However the response rates to analgesics were lower than those of the CM group. It is stated that patients with higher headache frequency and amount of analgesics consumption are at increased risk of developing CM+MOH [23]. The high severity and frequency of attacks in the CM+MOH group make the treatment of the disease complex, which may lead to increased analgesic use [24]. Therefore, special treatment strategies and further research for patients with CM+MOH are required.

The increased API scores and addiction severity in the CM+MOH group indicate a remarkable relationship between this group and addictive behavior. The increased craving and motivation for substance use in this group further emphasizes the intertwined nature of migraine and addictive behaviors. This raises the question of conceptualizing MOH not only as a secondary consequence of migraine, but also as a complex comorbidity with distinct psychological components [7, 25]. It is suggested that treatment strategies in MOH that advocate a comprehensive approach that addresses both headache symptoms and underlying addictive behaviors are necessary [7, 26]. High scores in anger management failure, lack of safe behavior, novelty-seeking behavior, impulsivity, depression, and anxiety in the CM+MOH group underscore the complex interplay of psychological factors in the emergence of medication overuse. Aggression is likely to be a common feature in chronic migraine and comorbid aggression may cause suicidality [27, 28]. Perozzo et al. (2005) reported that migraine patients showed a significantly higher level of angry temperament and angry reaction [28]. The Eurolight Project, which gathered population-based data from 6624 participants, states that depression and anxiety levels were found to be high in migraine patients, especially migraineurs diagnosed with MOH [29]. Consistent with the literature, our research findings suggest that it is necessary to integrate psychological assessments and interventions into the clinical management of CM+MOH. High scores in this group highlight the need to increase awareness of psychological health considerations in this patient population. MOH correlates with an increased risk for suicidal ideation and suicide attempts, which deserves attention from clinicians taking care of headache patients [30]. Addressing these factors will improve migraine outcomes and contribute to a more comprehensive improvement in overall well-being. Identifying specific psychological problems in CM patients with a medication overuse can be considered necesarry for creating targeted therapeutic interventions.

The considerably elevated MIDAS scores within the CM+MOH group suggest that this patient cohort experiences a notable impairment in daily functionality. Incorporating disability assessments into the comprehensive evaluation of MOH patients is crucial [31]. MOH has a significant negative impact on the personal, family, and social lives of patients and is associated with depression, anxiety, and stress. The adverse consequences of physical, emotional, and social dysfunction play significant roles in exacerbating the detrimental effects of MOH [31]. The observed deterioration in daily activities and quality of life may be critical in the emergence of medication overuse.

As indicated by our SSAS scores findings, the prominence of somatic symptoms, underscores the need for a holistic approach in the clinical management of CM patients who exhibit medication overuse that considers both psychological and somatic symptoms. Somatic amplification plays a significant role in migraine-related disability [16]. It is shown that migraineurs had a higher than average tendency to be aware of bodily sensations and there is a positive correlation between the SSAS scores and the frequency of headaches [16]. Although there was a significant difference in SSAS scores between the CM and control groups in our study, Yavuz et al. (2013) found that migraine patients showed a higher tendency towards somatization compared to the control group [16]. It can be said that this difference may be caused by the fact that migraine patients in Yavuz et al.'s (2013) study were not divided into CM and CM+MOH. The predictive value of craving and SSAS scores in our study indicates there is an meaningful interaction between psychological and somatic factors in choosing medication usage patterns. The significance of SSAS scores in the CM+MOH group highlights the central role of somatic amplification in guiding analgesic use. Individuals with migraines, who exhibit heightened awareness of unpleasant bodily sensations and a tendency to interpret ambiguous bodily feelings as abnormal or pathological, may perceive migraine attacks as more severe.Consequently, this tendency may prompt migraine sufferers to rely more on painkillers. Psychosomatic factors are important in chronic headache symptoms, especially tension-type headaches [32]. It has been reported that psychiatric disorders can also lead to MOH and chronic migraine [14]. Targeted interventions to address somatic symptoms in CM patients with an excessive amount of analgesic use seem to be necessary for the successful treatment of the disease. A multidisciplinary approach could prove beneficial for such patients. In addition to neurologists, psychiatrists, psychologists, nurses, and even social workers can play vital roles in helping MOH patients address and resolve a range of issues. [33]. The main psychological interventions as treatments for migration include relaxation training, cognitive behavioral therapy and biofeedback [34]. Furthermore, while we acknowledge the role of somatosensory amplification in the development of medication overuse headache, it's equally crucial to assess and address other psychiatric conditions like anxiety disorders and depression, which may influence similar symptoms. [16]. It is vital to conduct further research to elucidate the underlying mechanisms driving these relationships and to inform the development of more targeted treatment strategies for migraine patients with medication overuse.

This study has some limitations. The patients were recruited from the neurology outpatient clinic of a university hospital and only patients between the ages of 18 and 65 were included in the study. This raises concerns about generalizability. While neurologists confirmed responses on the instruments through face-to-face interviews, patients themselves completed the SSAS, SBQ, API, and API-C. Therefore, there may be concerns about reliability. Finally, as a cross-sectional study, only associations, rather than causal relationships, could be identified, and the findings should be interpreted cautiously.

## Conclusion

The present study demonstrated that the predictive capacity demonstrated by both craving levels and SSAS scores underscores the potential significance of these parameters in the context of medication overuse among individuals with migraines, as well as in the therapeutic intervention process. When formulating treatment strategies for migraineurs struggling with medication overuse, it is imperative to conduct a comprehensive evaluation of additional addiction-related attributes, alongside a thorough assessment of the psychosomatic complaints articulated by the patients.

## Data Availability

The data that supporting the findings of this study will be made available on request from the corresponding author.
